# Effects of the Positive Allosteric Modulator of Metabotropic Glutamate Receptor 5, VU-29, on Maintenance Association between Environmental Cues and Rewarding Properties of Ethanol in Rats

**DOI:** 10.3390/biom10050793

**Published:** 2020-05-20

**Authors:** Marta Marszalek-Grabska, Kinga Gawel, Dariusz Matosiuk, Ewa Gibula-Tarlowska, Joanna Listos, Jolanta H. Kotlinska

**Affiliations:** 1 Department of Experimental and Clinical Pharmacology, Medical University of Lublin, Jaczewskiego 8b, 20-090 Lublin, Poland; kinga.gawel@umlub.pl; 2 Department of Synthesis and Chemical Technology of Pharmaceutical Substances with Computer Modelling Lab, Medical University of Lublin, Chodzki 4a, 20-093 Lublin, Poland; dariusz.matosiuk@umlub.pl; 3 Department of Pharmacology and Pharmacodynamics, Medical University of Lublin, Chodzki 4a, 20-093 Lublin, Poland; ewa.gibula@umlub.pl (E.G.-T.); joanna.listos@umlub.pl (J.L.); jolanta.kotlinska@umlub.pl (J.H.K.)

**Keywords:** VU-29, mGlu5 receptor, positive allosteric modulator, conditioned place preference, ethanol, reward, memory

## Abstract

Metabotropic glutamate subtype 5 (mGlu5) receptors are implicated in various forms of synaptic plasticity, including drugs of abuse. In drug-addicted individuals, associative memories can drive relapse to drug use. The present study investigated the potential of the mGlu5 receptor positive allosteric modulator (PAM), VU-29 (30 mg/kg, i.p.), to inhibit the maintenance of a learned association between ethanol and environmental context by using conditioned place preference (CPP) in rats. The ethanol-CPP was established by the administration of ethanol (1.0 g/kg, i.p. ×10 days) using an unbiased procedure. Following ethanol conditioning, VU-29 was administered at various post-conditioning times (ethanol free state at the home cage) to ascertain if there was a temporal window during which VU-29 would be effective. Our experiments indicated that VU-29 did not affect the expression of ethanol-induced CPP when it was given over two post-conditioning days. However, the expression of ethanol-CPP was inhibited by 10-day home cage administration of VU-29, but not by first 2-day or last 2-day injection of VU-29 during the 10-day period. These findings reveal that VU-29 can inhibit the maintenance of ethanol-induced CPP, and that treatment duration contributes to this effect of VU-29. Furthermore, VU-29 effect was reversed by pretreatment with either MTEP (the mGlu5 receptor antagonist), or MK-801 (the N-methyl-D-aspartate-NMDA receptor antagonist). Thus, the inhibitory effect of VU-29 is dependent on the functional interaction between mGlu5 and NMDA receptors. Because a reduction in ethanol-associated cues can reduce relapse, mGlu5 receptor PAM would be useful for therapy of alcoholism. Future research is required to confirm the current findings.

## 1. Introduction

Addiction to ethanol and other drugs of abuse results in part from exceptionally strong, maladaptive associations formed between contextual cues and the rewarding properties of the drug [1,2,3]. These drug-associated contextual memories are long lasting, highly resistant to extinction, and contribute to the high rate of relapse in ethanol-dependent individuals, as well as the reinstatement of ethanol-seeking behavior [4,5,6,7,8].

Conditioned place preference (CPP) paradigm is an animal model used to study the rewarding effects of addictive drugs that require the formation of learning associations between reward and particular location [9,10]. Following pairings of a drug treatment with a specific environmental context, the expression of a CPP is assessed on a treatment-free test day. Therefore, CPP behavior ultimately involves acquisition, consolidation and retrieval (CPP expression) of stimulus–reward memory for generating an association between environmental stimuli and the affective state produced by a treatment [11,12].

The CPP paradigm also allows the studying of relapse to drug abuse after the extinction and reinstatement of CPP. The extinction procedure typically involves exposing animals to the previously drug-paired context in a drug-free state [13,14] until preference is no longer observed. Extinction is usually performed either by administering injections of a vehicle in both the original drug-paired and the original vehicle-paired chambers or by repeating CPP tests until preference is no longer observed. Thus, such a procedure should facilitate the extinction of cue–drug memories to reduce relapse [15].

Behavioral evidence indicates that the mechanism of the underlying extinction of conditioned memories involves establishing a new form of inhibitory learning in which the conditioned stimulus progressively loses its ability to evoke the conditioned response [16,17,18]. Following extinction, relapse can be modelled by the ability of a various precipitators (stress, drug-priming or drug-associated cues) to reinstate conditioned drug-seeking behavior [13,19,20]. However, many ethanol/drug users do not undergo the equivalent of extinction training via rehabilitation programs [21,22].

Alternatively, removing animals from drug-conditioning apparatus without providing extinction training provides a model of abstinence (withdrawal). Following withdrawal, conditioned drug-seeking behavior is elicited by environmental and contextual cues [23]. It should be noted that periods of withdrawal have actually been associated with subsequent increases in cue-induced drug seeking. This phenomenon was first observed in human drug users, and researchers [24,25,26] have confirmed this effect in preclinical experiments [27,28,29,30]. Although drug-seeking behavior decreases after more extended periods of abstinence, human drug users have been shown to exhibit increases in cue-related cravings during the first few months of withdrawal (40–60%) or even by 1 year (70–80%) [26,31,32]. Therefore, animal models that examine the effect of a period of abstinence on cue-induced drug-seeking possess implicit translational value.

The cellular basis of learning and memory and associated synaptic plasticity such as long-term potentiation (LTP) and long-term depression (LTD) of synaptic efficacy require glutamatergic transmission to occur [33,34]. In particular, the metabotropic glutamate subtype 5 (mGlu5) receptors are known to play an important role in learning and memory [1,35,36]. These receptors belong to group I mGlu receptors and are linked via scaffold proteins, including Shank and Homer, to N-methyl-D-aspartate (NMDA) receptors [37,38,39]. Through this mechanism, they are implicated in regulating the induction and maintenance of synaptic plasticity—the putative neurochemical basis of learning and memory [40].

In recent years, multiple mGlu5 receptor positive allosteric modulators (PAMs) have been identified and shown to have cognitive-enhancing effects in a number of animal models [41,42,43,44]. Previous studies have shown that mGlu5 PAMs enhance synaptic plasticity and cognitive function by the potentiation of the mGlu5-dependent regulation of NMDA receptor signaling (nonbiased mGlu5 PAMs). However, new mechanistic insights in the actions of mGlu5 PAMs suggest that the modulation of NMDA receptor is not crucial for the in vivo efficacy of mGlu5 PAMs. For instance, a recently discovered novel group of biased ligands of mGlu5 PAMs has been found that selectively potentiates the coupling of mGlu5 to G_∝q_ GTP-binding proteins and subsequently mobilizes intracellular calcium and activates protein kinase C and associated signaling pathways that lead either (via complex kinase cascade and Arc protein) to the internalization of α-amino-3-hydroxy-5-methyl-4-isoxazolepropionic acid (AMPA) receptors [45] or the activation of presynaptic type 1 cannabinoid (CB1) receptors. In turn, the activation of presynaptic CB1 receptors regulates γ-aminobutyric acid (GABA)/glutamate release [46,47]. Taking into account the above data, biased ligands of mGlu5 PAMs might have, being cognitive enhancers, a better safety profile than nonbiased mGlu5 PAMs that induce neurotoxicity associated with increased NMDA receptor currents [47,48].

Critically, mGlu5 receptors are distributed throughout the neural circuitry involved in reward-driven behaviors [49,50]. The activation of these receptors is involved in facilitating the extinction of drug-seeking behavior [3,51]. For these reasons, the mGlu5 receptors have received considerable attention in recent years as a potential therapeutic target for the treatment of drug addiction [37,39,40,52].

Our previous study indicated that the mGlu5 receptor PAMs, including VU-29, attenuated cognitive deficits induced by acute ethanol administration and ethanol withdrawal [53,54]. Taking into account that the brain regions and neuronal processes that underlie addiction overlap extensively with those that support cognitive function [2], we sought to determine whether VU-29, given during the abstinence/withdrawal period in home cages to ethanol-conditioned rats was able to affect the short/long-term maintenance of ethanol-induced CPP. To determine the mechanism of VU-29 effects, we used MTEP, an antagonist of mGlu5 receptors, and MK-801, a noncompetitive antagonist of NMDA receptors. Additionally, we investigated whether VU-29 given alone is able to induce rewarding effects in the CPP test. Moreover, the influence of VU-29 on the locomotor activity and anxiety-like behavior of rats was investigated to rule out such effects on the obtained results. Answering these questions is equivalent to deciding whether VU-29 might be useful to disrupt associative memories that can drive relapse to drug use in drug-addicted individuals.

## 2. Materials and Methods

### 2.1. Animals

Male naïve Wistar rats (HZL, Warsaw, Poland) weighing 200–250 g at the initiation of the experimental procedure (age of 8–9 weeks) were used in our experiments. The rats were housed four per cage with a standard diet (Agropol, Motycz, Poland) and water *ad libitum*, and kept under 12-h light/dark cycle and controlled temperature (22 ± 2 °C). The rats were adapted to the laboratory conditions and were handled for at least 1 week before the experiments. Each experimental group consisted of 8–10 rats. All behavioral studies were performed between 9:00 a.m. and 5:00 p.m. The experimental protocols and housing conditions were performed according to the National Institute of Health Guidelines for the Care and Use of Laboratory Animals, the European Community Council Directive of November 2010 for Care and Use of Laboratory Animals (Directive 2010/63/EU), and were approved by the 1st Local Ethics Committee, Lublin, Poland (No. 67/2017).

### 2.2. Drugs

Ethanol (95%, *w*/*v*, Polmos, Poznan, Poland) was diluted in saline (0.9% NaCl) to a concentration of 10% (*w*/*v*) and administered intraperitoneally (i.p.) at the dose of 1.0 g/kg. This ethanol dosage regimen was established in our preliminary study, wherein the dose of 1.0 g/kg conditioned place preference in rats. MK-801 (0.1 mg/kg; Sigma-Aldrich, St. Louis, MO, USA) and MTEP (5 mg/kg; Sigma-Aldrich, St. Louis, MO, USA) were dissolved in saline and given i.p. Ethanol, MK-801 and MTEP were given in a volume of 2 mL/kg. The selective mGlu5 positive allosteric modulator N-(1,3-diphenyl-1H-pyrazolo-5-yl)-4-nitrobenzamide (VU-29) (donated by the Department of Synthesis and Chemical Technology of Pharmaceutical Substances with Computer Modelling Lab, Medical University, Lublin, Poland) was dissolved in a vehicle consisting of 10% Tween-80 (Sigma) in saline and given at the dose of 30 mg/kg, i.p., in a volume of 1 mL/kg. Injection timepoint and the dose for VU-29 were chosen based on our previous study [53].

### 2.3. CPP Apparatus

In the present study, eight wooden conditioning chambers (60 × 35 × 30 cm) were used. Each of these consisted of two large compartments (25 × 35 cm) separated by removable guillotine doors from a small central gray area (10 × 10 cm). The walls of the two large compartments differed in color, with one having white walls, while the other one was black. To provide a tactile difference between the compartments, one of the compartments (white) had a smooth floor, while the other one (black) had a grid floor. The whole apparatus was cleaned thoroughly between each test procedure to neutralize the odor trails, and then wiped with dry paper towels. The boxes were kept in a soundproof room with a neutral masking noise and with a dim 40 lx illumination. Data were collected by cameras (Karnet, Lublin, Poland) located above each chamber and were automatically sent to a computer for storage and analysis.

### 2.4. CPP Procedure

The CPP procedure was based on methods described previously [55,56] with minor modifications. The CPP procedure (unbiased design) consisted of six different phases: habituation (1 day), pre-test (1 day), conditioning (10 days), followed by Test 1 and withdrawal (2 or 10 days), followed by Test 2 (1 day). The amount of time spent in each compartment was measured during the pre-test phase. These results were used to separate animals into groups with approximately equal biases for each side. Moreover, an appropriate control group was used that underwent the same CPP procedure as the drug-treated rats.

*Habituation and pre-conditioning test*: The first and second phases of the experiment (days 1–2) were aimed at assessing primary place preference and consisted of measuring the time of residence in the two areas of apparatus for 15 min. During these phases, the animals were placed separately in the central, small gray area with the guillotine doors removed to allow access to the entire apparatus. On the second day (i.e., the pre-conditioning test), the time spent by the rats in each of the two large compartments was measured in order to determine an initial preference that was equal to our unbiased experimental design. In the particular experimental setup that we used in our study, the animals did not show a significant preference for either of the compartments during this phase. No injections were given to rats during this phase.

*Conditioning*: The third phase of the experiment consisted of two per day 30-min morning and afternoon sessions for 10 days. Here, the rats were randomly assigned to control and ethanol groups. In the morning session, the animals were injected with saline and confined in one compartment. The afternoon session was conducted with an interval of at least 4 h. In the afternoon session, control groups were injected with saline, ethanol groups received ethanol (1.0 g/kg, 10% *w*/*v*, i.p.) and were conditioned via the opposite compartment. Guillotine doors, separating the two areas, were closed. Injections were administered immediately before confinement in one of the two large compartments. A dose of 1.0 g/kg ethanol was chosen for conditioning because it produces reliable CPP in rats after 10 days of conditioning. The neutral zone was never used during conditioning and was blocked by guillotine doors. The post-conditioning test (Test 1—expression of CPP) was conducted 24 h after the last conditioning session. In this, the rats were given free access to the experimental compartments for 15 min, during which the amount of time spent in each of the two large compartments was animals entered a withdrawal phase in their home cages (see the Experimental Procedure).

### 2.5. Experimental Procedure

#### 2.5.1. Experiment 1: The Effect of VU-29 on the Short-Term Maintenance of Ethanol-Induced CPP

**Figure 1:**
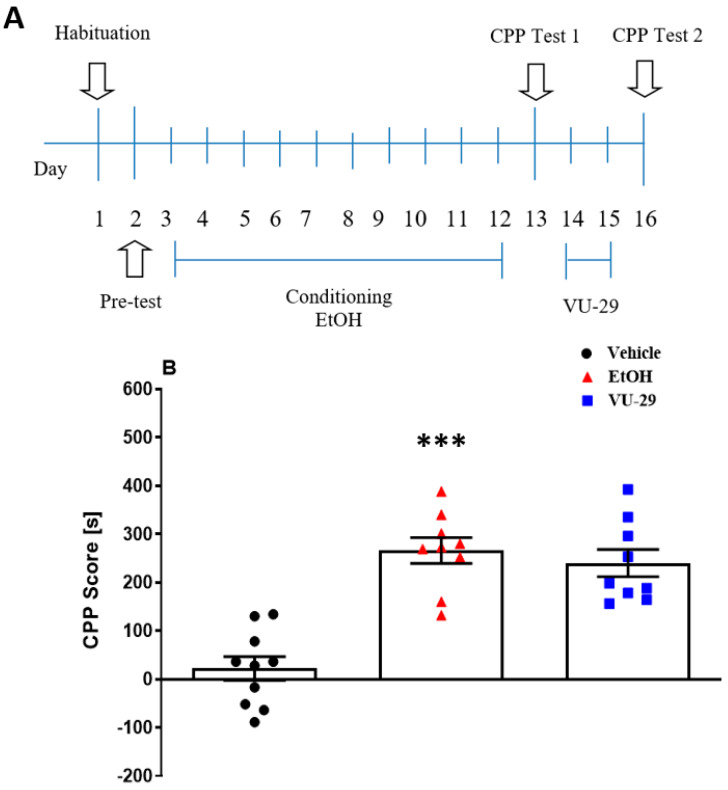
Experimental design for the effect of VU-29 on short-time maintenance of ethanol-induced CPP (**A**). Data are shown as post-conditioning minus pre-conditioning time (s) spent in the drug-associated compartment in the CPP Test 2 (**B**). Dots represent individual measurements, the central horizontal mark is the mean, and error bars represent SEM. *** *p* < 0.001 vs. vehicle.

The study (see Figure 1A) was designed to ascertain if early post-conditioning treatments of VU-29 disrupted the short-term maintenance of ethanol-induced CPP. Thus, one day after the pre-conditioning test, the rats were conditioned with ethanol over 10 days, with two 30 min conditioning sessions each day. In the morning, the rats were given vehicle and immediately placed in one CPP chamber and 4 h later, in the afternoon session, the other chamber was paired with ethanol (1.0 g/kg, 10% *w*/*v*, i.p.). One day after the final conditioning session, rats were given the 15 min, drug-free Test 1. Then, 24 h later, ethanol-conditioned rats were injected with VU-29 (30 mg/kg, i.p.) or vehicle in the home cage (withdrawal), once-daily for two days. One day after the VU-29/vehicle injection, drug-free Test 2 was performed.

#### 2.5.2. Experiment 2: The Effect of VU-29 on the Long-Term Maintenance of Ethanol-Induced CPP

**Figure 2:**
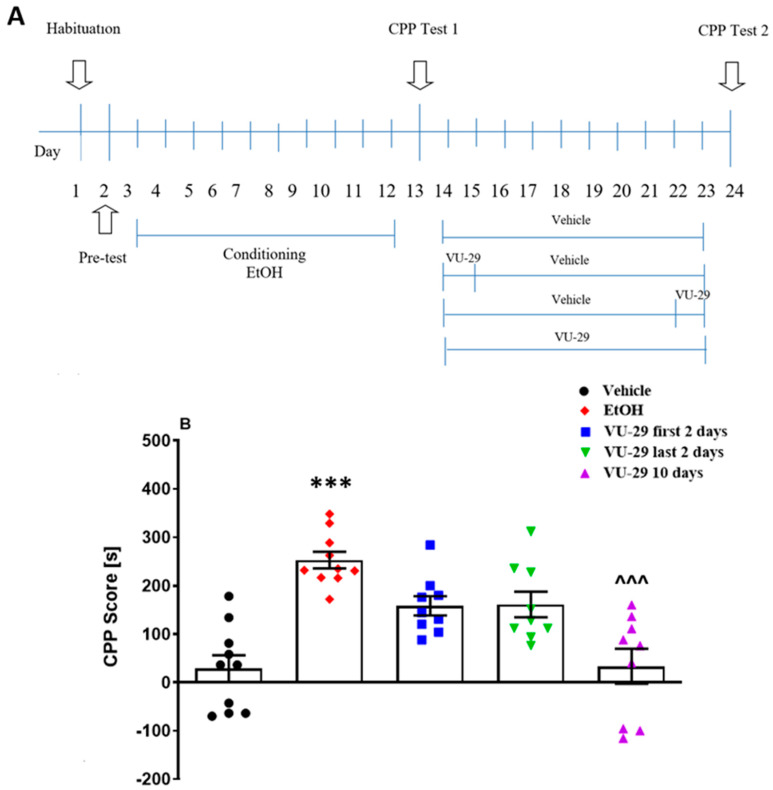
Experimental design for the effect of VU-29 administered in three different combinations on long-time maintenance of ethanol-induced CPP (**A**). Data are shown as post-conditioning minus pre-conditioning time (s) spent in the drug-associated compartment in the CPP Test 2 (**B**). Dots represent individual measurements, the central horizontal mark is the mean, and error bars represent SEM. *** *p* < 0.001 vs. vehicle; ^^^ *p* < 0.001 vs. ethanol-treated group.

The study (see Figure 2A) was designed to determine the effect of VU-29 on the long-term maintenance of ethanol-induced CPP. One day after the pre-conditioning test, the rats were conditioned over 10 days, similar to the method described above. One day after the final conditioning session, the rats were given the 15 min, drug-free Test 1. One day later, the rats received either: (1) 10 days of vehicle, (2) 10 days of VU-29 (30 mg/kg, i.p.), (3) two days of VU-29 (30 mg/kg, i.p.) followed by eight days of vehicle, or (4) eight injections of vehicle followed by two days of VU-29 (30 mg/kg, i.p.) in their home cages (withdrawal). One day after the last VU-29/vehicle injection, drug-free Test 2 was performed.

#### 2.5.3. Experiment 3: The Influence of MK-801 and MTEP on the Effect of VU-29 on the Long-Term Maintenance of Ethanol-Induced CPP

**Figure 3:**
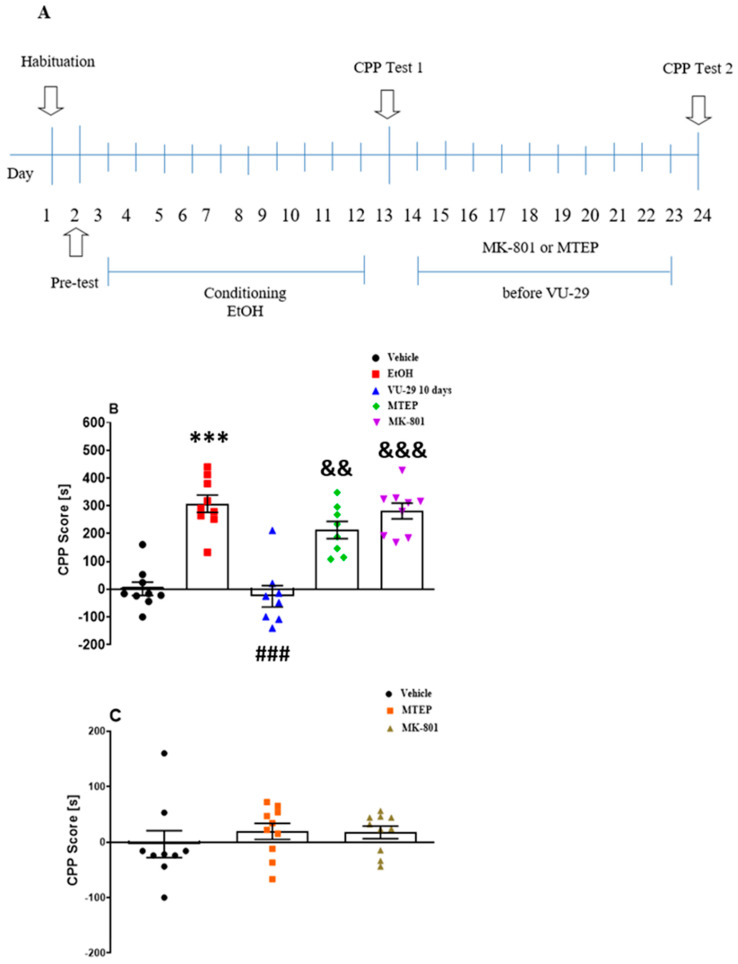
Experimental design for the influence of MK-801 and MTEP on the effect of VU-29 on the maintenance of ethanol-induced CPP (**A**). Data are shown as post-conditioning minus pre-conditioning time (s) spent in the drug-associated compartment in the CPP Test 2 (**B**). The influence of MK-801 and MTEP on CPP score in vehicle-treated rats in the CPP Test 2 (**C**). Dots represent individual measurements, the central horizontal mark is the mean, and error bars represent SEM. *** *p* < 0.001 vs. vehicle; ^###^ *p* < 0.001 vs. ethanol-treated group; ^&&^ *p* < 0.01, ^&&&^ *p* < 0.001 vs. 10 Day VU-29-treatment.

Experiment 3 (see Figure 3A) was designed to assess the influence of MK-801 (0.1 mg/kg, i.p.), or MTEP (5 mg/kg, i.p.), on the effect of VU-29 (30 mg/kg, i.p.) on the long-term maintenance of ethanol-induced CPP. In this set of experiments, rats received MK-801 or MTEP, respectively, 30 and 15 min before each of 10-day injection of VU-29. The procedure was similar to the method described above. Injection time and doses of MK-801 and MTEP were based on our previous study [57]. To investigate the influence of MK-801 and MTEP on CPP score in vehicle-treated rats, a separate group of animals received MK-801 or MTEP for 10 days as described above.

#### 2.5.4. Experiment 4: The Effect of VU-29 on Rat Behavior in the CPP Procedure

**Figure 4:**
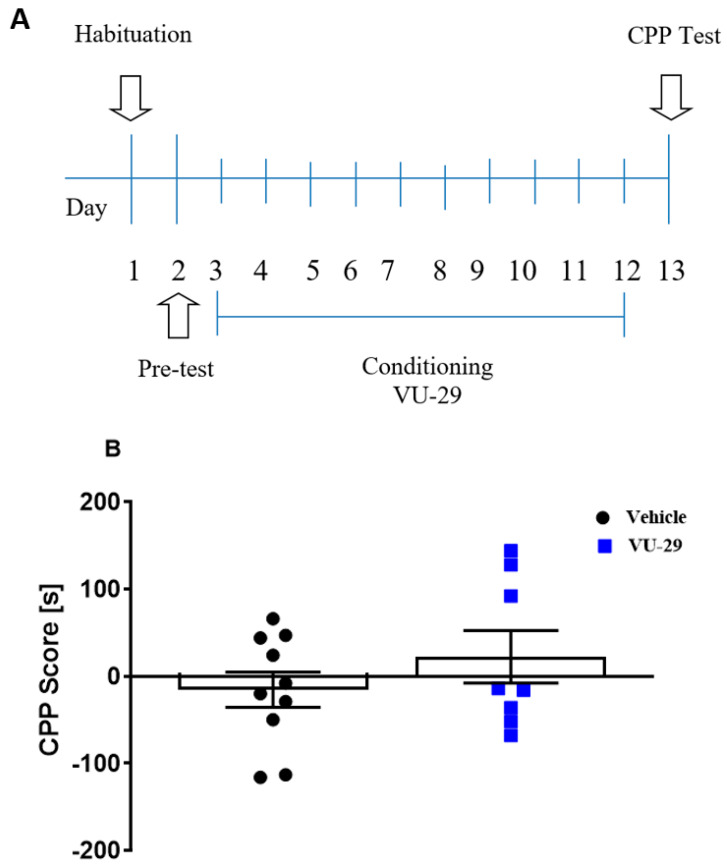
Experimental design for the effect of VU-29 on rat behavior in the CPP procedure (**A**). Data are shown as post-conditioning minus pre-conditioning time (s) spent in the drug-associated compartment in the CPP Test (**B**). Dots represent individual measurements, the central horizontal mark is the mean, and error bars represent SEM.

The experiment (see Figure 4A) was designed to determine if VU-29 was rewarding or aversive. One day after the pre-conditioning test, rats were conditioned over 10 days, with two 30 min-long conditioning sessions each day. In the morning, the rats were given saline and immediately placed in one CPP chamber and 4 h later, in the afternoon session, the other chamber was paired with VU-29 (30 mg/kg, i.p.), given 20 min before session. One day after the final conditioning session, rats were given a 15 min, drug-free CPP test. In Experiment 4, the locomotor activity of individual rats was measured on the first and last day of conditioning with VU-29, to rule out the possibility that the observed changes may be due to motor disturbances.

### 2.6. Elevated Plus-Maze Experiment

#### 2.6.1. Elevated Plus-Maze Apparatus

The plus-shaped maze was made of wood and positioned on a height of 50 cm above the floor, in a quiet laboratory surrounding. Two opposite arms were open (50 × 10 cm) and the other two were enclosed with walls (50 × 10 × 40 cm). The experiments were carried out in a quiet, darkened room with a constant light of 100 lx, located 80 cm above the maze, and directed towards the apparatus. The experiment was initiated by placing the rat in the center of the plus-maze, facing an open arm, after which the number of entries, the time spent in each of the two arms, the distance traveled in open arms, as well as the total distance traveled were recorded for a period of 5 min. Each “arm entry” was recorded when the rat entered the arm with all four paws. The maze was carefully cleaned with tap water after each test session [58,59]. The experiments were videotaped and all recordings were made manually by a highly trained experimenter, blind to treatment groups.

#### 2.6.2. Elevated Plus-Maze Procedure

Separate groups of animals were used to investigate the influence of VU-29 on rat behavior in the elevated plus-maze test. Here, VU-29 was given once daily for 10 days in their home cages. The potential anxiety-like effects of VU-29 (30 mg/kg, i.p.) for each rat was measured 20 min after the last VU-29 administration as: a) the number of entries into the open arms—as a percent of the total number of entries into both open and closed arms (% open arm entries); b) the time spent in the open arms-as a percent of total time spent on exploring open and closed arms (% time in open arms). Furthermore, the locomotor activity of animals was evaluated as the total number of entries into the both open and closed arms of the apparatus.

### 2.7. Locomotor Activity

The horizontal activity boxes (Porfex, Bialystok, Poland) for rats are Plexiglas square chambers (60 cm each side) located in a sound-attenuated experimental room, under moderate illumination (5 lx). Horizontal activity (distance traveled in meters) was measured by two infrared light-sensitive photocells located 45 and 100 mm above the floor. The locomotor activity test was conducted on the first and last day of VU-29 administration. Locomotor activity was measured in the group of animals that received MK-801 and MTEP before VU-29 to assess the influence on these compounds on the rat locomotor activity. In addition, the number of crossings, from one compartment to another through the central grey area was recorded. This is presented in the tables as a readout of animal locomotor activity for each experiment. The animals were moved to the locomotor activity boxes and locomotor activity was assessed for a total period of 15 min. To avoid stress reactions, the animals were habituated to the apparatus 2 days before the experiment (15 min).

### 2.8. Statistical Analysis

CPP data are expressed as individual measurements (i.e., dots), the means ± standard error of the mean (SEM). The preference scores were calculated as follows: the post-conditioning minus the pre-conditioning time intervals, spent at the drug-associated compartment. The statistical significance of drug effects in the tests was assessed by one-way analysis of variance (ANOVA), and the significance of difference between individual groups was determined by Tukey’s *post hoc* test. Specific paired comparison was performed with Student’s *t*-test when necessary. Statistical significance was set at *p* < 0.05. All data were performed using GraphPad Prism 6.0 Software, San Diego, California, USA.

## 3. Results

### 3.1. Experiment 1: The Effect of VU-29 on the Short-Term Maintenance of Ethanol-Induced CPP

Figure 1A shows the experimental design in which the rats first acquired CPP as measured in the drug-free Test 1, subsequently given two daily trials (VU-29 was given in home cages for 2 days) and, afterwards, tested in the drug-free Test 2. One-way ANOVA revealed significant differences among the assessed groups on CPP Test 1 [F(2,27) = 12.35, *p* < 0.001, Figure S1] and CPP Test 2 [F(2,27) = 8.14, *p* < 0.001, Figure 1B]. Tukey’s *post-hoc* analysis showed that the animals spent more time in the drug-paired compartment in Test 1 (*p* < 0.001, Figure S1) and in Test 2 (*p* < 0.001, Figure 1B) following ethanol conditioning, as compared to the vehicle group. VU-29 given once daily in the home cages for two days did not significantly reduce the preference for the ethanol-paired chamber. Thus, two days of VU-29 administration during the early post-conditioning phase (withdrawal) did not disrupt the short-term maintenance of ethanol-induced CPP. In addition, as shown in Table 1, the used drugs had no effect on the locomotor activity of rats in Test 2.

### 3.2. Experiment 2: The Effect of VU-29 on the Long-Term Maintenance of Ethanol-Induced CPP

To ascertain if increasing the number of VU-29 (30 mg/kg, i.p.) treatments could disrupt the long-term maintenance of ethanol-induced CPP, 10 home-cage treatments were administered. Figure 2A shows the experimental design. One-way ANOVA test revealed significant differences among the group results of CPP Test 1 (F(4,64) = 12.69, *p* < 0.0001, Figure S2) and CPP Test 2 (F(4,64) = 7.51, *p* < 0.0001, Figure 2B). Tukey’s *post-hoc* analysis showed that the animals spent more time in the drug-paired compartment in Test 1 (*p* < 0.001, Figure S2) and in Test 2 (*p* < 0.001, Figure 2B), following ethanol conditioning, as compared to the vehicle group. VU-29 given for 10 days in home cages disrupted ethanol-induced CPP. In Test 2, the animals spent significantly less time in the drug-paired compartment as compared to the ethanol-treated group (*p* < 0.001).

To determine if the ability of VU-29 to antagonize the maintenance of ethanol-induced place preference reflected processes that occurred only at the beginning or at the end of the 10 days VU-29 treatment period, VU-29 injections were given on the first 2 days or last 2 days of the 10 days VU-29 treatment protocol. Neither early nor late VU-29 treatments disrupted CPP. While the first 2 days and last 2 days VU-29 treatment slightly reduced preference for the ethanol-paired chamber, CPP was retained with sufficient magnitude to preserve significance (*p* < 0.001). Thus, treatment duration, and not the post-conditioning phase in which the VU-29 was administered, was critical for inhibiting the maintenance of ethanol-induced CPP. In addition, as shown in Table 2, the used drugs had no effect on the locomotor activity of rats in Test 2.

### 3.3. Experiment 3: The Influence of MK-801 and MTEP on the Effect of VU-29 on the Long-Term Maintenance of Ethanol-Induced CPP

To assess the influence of MK-801 (0.1 mg/kg, i.p.), a noncompetitive antagonist of NMDA receptors or MTEP (5 mg/kg, i.p.), an antagonist of mGlu5 receptors, on the 10 once-daily treatment of VU-29 (30 mg/kg, i.p.) over the post-conditioning period, the rats received MK-801 or MTEP, respectively, 30 and 15 min before each of 10 day injections of VU-29 in their home cages. Figure 3A shows the experimental design. One-way ANOVA test on CPP Test 1 (F(4,42) = 14.04, *p* < 0.0001, Figure S3A) and CPP Test 2 (F(4,42) = 8.42, *p* < 0.0001, Figure 3B) revealed significant differences among the groups. A *post-hoc* analysis (Tukey–Kramer test) showed that the animals spent more time in the drug-paired compartment in CPP Test 1 (*p* < 0.001, Figure S3A) and in CPP Test 2 (*p* < 0.001, Figure 3B). Furthermore, once daily treatment of VU-29 for 10 withdrawal days in the home cage disrupted ethanol-induced CPP (*p* < 0.001). This effect was reversed by MTEP (*p* < 0.01) and MK-801 (*p* < 0.001), respectively.

To assess the influence of MK-801 and MTEP on CPP score in vehicle-treated rats, a separate group of animals received MK-801 or MTEP for 10 days as described above. One-way ANOVA of the results of Test 1 (F(2,25) = 0.97, *p* > 0.05, Figure S3B) and Test 2 (F(2,25) = 0.59, *p* > 0.05, Figure 3C) did not reveal significant differences among the groups. In addition, as shown in Table 3, the used drugs had no effect on the locomotor activity of rats in Test 2.

### 3.4. Experiment 4: The Effect of VU-29 on Rat Behavior in the CPP Procedure

Figure 4A shows the experimental design of the effect of VU-29 on rat behavior in the CPP procedure. Ten days of VU-29 (30 mg/kg, i.p.) administration did not induce a chamber bias (t = 0.570, *p* > 0.05; Figure 4B), suggesting that VU-29 is neither rewarding nor aversive. In addition, as shown in Table 4, the used drugs had no effect on the locomotor activity of the rats in CPP Test. Furthermore, locomotor activity measured on the first and last day of conditioning with VU-29 revealed that behavioral response to VU-29 remained unchanged throughout the 10 days’ protocol. This indicates that neither sensitization nor tolerance occurred as a result of the 10 once-daily treatment (see Table 5).

### 3.5. Experiment 5: Effect of VU-29 on Plus-Maze Performance

VU-29 (30 mg/kg, i.p.) did not change the number of entries into the open arms (t = 1.370, *p* > 0.05), the time spent by rats in the open arms (t = 0.852, *p* > 0.05), the number of total entries into the both open and closed arms (t = 0.236, *p* > 0.05), the distance traveled in open arms (t = 0.534, *p* > 0.05) and the total distance traveled (t = 0.805, *p* > 0.05) in the plus-maze apparatus during the 5 min observation sessions (see Table 6). Thus, treatment with VU-29 had no effect on anxiety-like behavior as compared to the control group.

### 3.6. Experiment 6: The Locomotor Activity Test

The locomotor activity test indicated that MK-801 and MTEP given before every VU-29 administration during the 10-day withdrawal period did not have any impact on locomotion in Test 2 (F(4,42) = 0.71, *p* > 0.05; see Table 7).

## 4. Discussion

Our study revealed that the mGlu5 receptor PAM, VU-29 administered to rats in the neutral environment of the home cage, was sufficient to diminish the previously expressed preference for the ethanol-paired chamber. However, there was a time-dependent relationship between VU-29 administrations and the maintenance of ethanol-induced CPP. In our study, the maintenance of previously acquired ethanol CPP was disturbed only after 10 days of VU-29 administrations. Furthermore, VU-29 alone had neither an aversive nor rewarding properties in the CPP test. Thus, a particularly important observation is that mGlu5 PAM modulation with VU-29 attenuated maladaptive memories involved in ethanol relapse following withdrawal, and that this VU-29 effect is dependent on the time of its administration.

Relapse to drug use can occur after prolonged abstinence and is often precipitated by exposure to craving-provoking drug-associated cues [28]. Based on the clinical observation, this phenomenon increases during early abstinence and remains elevated for an extended time period [24,60]. Such a phenomenon, termed “incubation of drug craving” [27], has been observed in self-administration procedure in rats with a history of ethanol exposure [61]. In our study, ethanol (1.0 g/kg, i.p., once daily for 10 days) conditioning induced a preference for the ethanol-paired chamber in the CPP procedure on the test day in rats. This ethanol effect was not affected by home cage injections of saline because the repeated exposure of rats to the conditioning environment following the 2- and 10-day abstinence interval produced the conditioned reward. Thus, ethanol-induced conditioned memory lasted at least 10 days after the last conditioning session. We did not measure the influence of ethanol withdrawal on incubation craving, however, published data indicate that CPP paradigm is applicable to the model of such phenomenon [30,62].

Our experiments revealed that the long-term maintenance of mnemonic association between the rewarding effects of ethanol and the ethanol-paired context could be disrupted by 10 once-daily injections of 30 mg/kg VU-29. Herein, we have indicated that long-term (10 days) but not short-term (2 days) stimulation of mGlu5 receptors by VU-29 (injections in animals home cages) was sufficient to inhibit the maintenance of previously established ethanol-induced CPP. Furthermore, this effect was not dependent on the duration of withdrawal period, but rather on the number of VU-29 injections because VU-29 injection during the first or last 2-days of the 10-day withdrawal period did not change the responding behavior for environments associated with ethanol reward. Therefore, our findings indicate that relatively sustained and long-lasting VU-29-induced changes in neuronal signaling were necessary to disrupt CPP memory maintenance (a time-dependent effect).

The mechanisms underlying the action of mGlu5 receptor PAM on the maintenance of associative memories are unknown. Still, Besheer and colleagues indicated [63,64] that mGlu5 receptors activity in the nucleus accumbens (a central component of reward circuitry of the brain) is necessary and sufficient for the full expression of the interoceptive effects of alcohol. In our study, VU-29 given alone did not show any effect on emotion and motivation (10 days administration of VU-29 did not induce either rewarding or aversive effects) in CPP procedure. However, it can be hypothesized that treatment with the mGlu5 receptor PAM during the ethanol withdrawal altered the rats’ interoceptive state so that it is different from the state that is typically experienced during ethanol-CPP sessions. Thus, targeting mGlu5 may be useful, for example, for ameliorating anhedonia and dysphoria following the cessation of drug/ethanol intake [46].

Despite this inference, another interpretation of the present result is possible. The CPP paradigm is a task that requires behavioral flexibility [65]. Chronic ethanol exposure alters cortex and hippocampus function and results in cognitive deficits during withdrawal. These deficits may contribute to impaired behavioral flexibility and to the inability to reverse ethanol-context associations, contributing to relapse [53,54,66]. Our previous results show that ADX-47273, the mGlu5 receptor PAM, attenuated the ethanol withdrawal-induced deficits in cognitive flexibility [54]. Herein, behavioral flexibility involves new learning about changes in response/reward contingencies, and this allows rats to adapt to situations in which reward and goals were changed. Thus, the behavioral consequences of repeated VU-29 administration measured in the current study may be due to long-term adaptations that occur as a consequence of repeated VU-29 treatment. It is also possible that the 10-day VU-29 administration is a critical window of vulnerability (when stored memory can be weakened) [67] for disrupting the maintenance of ethanol-induced CPP (i.e., time-dependent effect). This process required permanent changes in the expression of signaling molecules associated with the activation of mGlu5 receptors [68] and probably the indirect stimulation of the NMDA receptors [69] that have been shown to be involved in various aspects of learning. In our previous experiments, ethanol withdrawal rats show an up-regulation of mGlu5 receptors and NR2B subunit of NMDA receptor. Here, mGlu5 PAMs, including VU-29, decreased these effects in brain structures such as the prefrontal cortex and hippocampus that are involved in memory processes [53,54].

Indeed, recent evidence has shown that the allosteric modulation of mGlu5 receptors affects NMDA receptor activity and this effect was inhibited by MK-801, a noncompetitive antagonist of NMDA receptors [70]. In our study, VU-29-induced inhibition of the maintenance of ethanol-induced CPP was reversed by MK-801 and MTEP (an antagonist of mGlu5 receptors). Although these data may not sound convincing enough, because MTEP can *per se* reduce ethanol-induced CPP [57,71,72] and MK-801 has been found to potentiate the reinstatement of ethanol-conditioned cues [73], nevertheless, published data support the notion that both of these receptors are involved in the VU-29 effects [53,54] and in VU-29-induced LTP in the hippocampal CA1 region in vitro [41]. Furthermore, MTEP or CDPPB (mGlu5 receptor PAM) decreased drug-seeking in response to cocaine-associated cues after prolonged abstinence. However, repeated treatment with MTEP impairs working memory, while CDPPB has no effect on performance [74]. It is rather difficult to determine how two mechanistically different pharmacological compounds can exert the same behavioral effects to reduce drug-seeking, but it seems that the mGlu5 receptor is important for the maintenance of cue and for contextual information associated with drug seeking.

Current data have revealed that VU-29, a mGlu5 PAM, is able to enhance hippocampal LTP by a mechanism that is not dependent on the potentiation of mGlu5 modulation of the NMDA receptor. Thus, VU-29 potentiates mGlu5 receptors in CA1 hippocampal pyramidal cells to stimulate the production and release of endocannabinoids (eCBs), which, in turn, act on CB1 receptors on neighboring interneuron terminals and decrease GABA release. This disinhibition could reduce inhibitory control of hippocampal CA1 pyramidal cells and subsequently facilitate LTP induction at SC-CA1 synapses. In contrast, CB1 inhibitors are able to block LTP and disrupt memory [47]. Therefore, CB1 antagonists [75] and mGlu5 PAMs could affect the ethanol-induced context-dependent memory by the modulation (blockade or facilitation) of LTP processes. However, further research is needed to support the role of CB1 receptor in the effects of VU-29.

On the other hand, VU-29, a mGlu5 receptor PAM, might change the response to drug-associated cues by alleviating negative withdrawal symptoms that can induce relapse. Indeed, published data indicate that systemic treatment with CDPPB exacerbates the effect of ethanol withdrawal on behavioral despair (anxiety- and depressive-like behavior), but only in adult mice [76]. Adolescent animals showed minimal response to CDPPB. Furthermore, this compound was modestly anxiogenic in both alcohol- and water-drinking mice. Our current study showed that VU-29, an analog of CDPPB, did not possess an anxiolytic/anxiogenic-like behavior *per se* and did not have an impact on locomotion. In actuality, these two compounds differ in their behavioral effects, although CDPPB also improves learning after alcohol administration [77].

In conclusion, our study, indicates for the first time that the maintenance of ethanol CPP is mitigated by repeated mGlu5 receptor PAM, VU-29 administration in the home cage. This effect requires long-term VU-29 administration. Thus, our outcome may suggest that permanent changes in glutamate neurotransmission during ethanol withdrawal are needed to disrupt the memory associated with the environment and drug administration. Such a VU-29 effect probably results from the engagement of mGlu5 and NMDA receptors. However, the usefulness of the mGlu5 receptor PAM as a treatment for ethanol abuse and cue-elicited relapse prevention needs further investigation.

## Figures and Tables

**Table 1:** The effect of 2 days VU-29 administration to ethanol-conditioned rats on locomotor activity measured as the number of crossings from one compartment to another during 15 min of Test 2. Results are expressed as mean ± SEM.

Group	Number of Crossings (15 min)	*p* Value	Number of Animals
Vehicle	51.20 ± 2.79	*p* > 0.05	10
EtOH	47.30 ± 2.95	*p* > 0.05	9
VU-29	48.50 ± 3.17	*p* > 0.05	9

**Table 2:** The effect of VU-29 administration to ethanol-conditioned rats on locomotor activity measured as the number of crossings from one compartment to another during 15 min of Test 2. Results are expressed as mean ± SEM.

Group	Number of Crossings (15 min)	*p* Value	Number of Animals
Vehicle	53.00 ± 2.73	*p* > 0.05	10
EtOH	50.50 ± 2.44	*p* > 0.05	10
VU-29 first 2 days	46.70 ± 2.87	*p* > 0.05	9
VU-29 last 2 days	48.80 ± 3.49	*p* > 0.05	9
VU-29 10 days	49.10 ± 2.64	*p* > 0.05	9

**Table 3:** The influence of MK-801 and MTEP on the effect of VU-29 administration to ethanol-conditioned rats on locomotor activity measured as the number of crossings from one compartment to another during 15 min of Test 2. Results are expressed as mean ± SEM.

Group	Number of Crossings (15 min)	*p* Value	Number of Animals
Vehicle	54.10 ± 2.27	*p* > 0.05	9
EtOH	51.80 ± 2.54	*p* > 0.05	9
VU-29 10 days	48.90 ± 2.45	*p* > 0.05	8
VU-29 + MTEP	53.10 ± 3.35	*p* > 0.05	8
VU-29 + MK-801	49.50 ± 2.47	*p* > 0.05	9
MTEP	51.70 ± 3.27	*p* > 0.05	10
MK-801	48.50 ± 2.41	*p* > 0.05	10

**Table 4:** The effect of VU-29 administration on locomotor activity as measured as the number of crossings from one compartment to another during 15 min of Test 2. Results are expressed as mean ± SEM.

Group	Number of Crossings (15 min)	*p* Value	Number of Animals
Vehicle	49.10 ± 2.55	*p* > 0.05	10
VU-29	52.10 ± 2.83	*p* > 0.05	8

**Table 5:** The effect of VU-29 on the locomotor activity of rats as measured on the first and last day of conditioning, and recorded as distance (m) traveled in 15 min. Results are expressed as mean ± SEM.

Group	Mean Distance Traveled (m)		*p* Value	Number of Animals
	1st Day of Conditioning	10th Day of Conditionig		
Vehicle	56.63 ± 3.58	49.79 ± 4.73	*p* > 0.05	10
VU-29	53.26 ± 4.68	54.14 ± 4.21	*p* > 0.05	8

**Table 6:** The effect of VU-29 on rat behavior in the plus-maze apparatus. The number of entries into the open arms (A), the time spent by rats in the open arms (B), the number of total entries into the both open and closed arms (C), the distance traveled in open arms (D), the total distance traveled (E). Results are expressed as mean ± SEM.

Parameter Measured	Group/Number of Animals		*p* Value
	Vehicle/11	VU-29/9	
(A) Open arms entries (%)	36.76 ± 4.62	44.28 ± 2.97	*p* > 0.05
(B) Time in open arms (%)	30.57 ± 3.47	35.63 ± 4.82	*p* > 0.05
(C) Number of total entries	9.13 ± 0.79	9.37 ± 0.71	*p* > 0.05
(D) Distance traveled in open arms (m)	4.69 ± 2.74	4.65 ± 2.47	*p* > 0.05
(E)Total distance traveled (m)	11.72 ± 2.34	10.34 ± 2.52	*p* > 0.05

**Table 7:** The influence of MK-801 and MTEP on the effect of VU-29 on the locomotor activity of rats recorded as distance (m) traveled in 15 min. Results are expressed as mean ± SEM.

Group	Mean Distance Traveled (m)	*p* Value	Number of Animals
Vehicle	23.40 ± 3.50	*p* > 0.05	9
EtOH	24.50 ± 2.93	*p* > 0.05	9
VU-29 10 days	21.20 ± 2.82	*p* > 0.05	8
VU-29 + MTEP	25.88 ± 2.39	*p* > 0.05	8
VU-29 + MK-801	20.38 ± 2.58	*p* > 0.05	9

## Supplementary Materials

The following are available online at https://www.mdpi.com/2218-273X/10/5/793/s1, Figure S1: The effect of VU-29 on short-time maintenance of ethanol-induced CPP in the CPP Test 1, Figure S2: The effect of VU-29 administered in three different combinations on long-time maintenance of ethanol-induced CPP in the CPP Test 1, Figure S3A: The influence of MK-801 and MTEP on the effect of VU-29 on the maintenance of ethanol-induced CPP in the CPP Test 1, Figure S3B: The influence of MK-801 and MTEP on CPP score in vehicle-treated rats in the CPP Test 1.
