# Supplementary material for: Effects of the Positive Allosteric Modulator of Metabotropic Glutamate Receptor 5, VU-29, on Maintenance Association between Environmental Cues and Rewarding Properties of Ethanol in Rats
Source: Biomolecules. 2020 May 20;10(5):793. doi: 10.3390/biom10050793 (PMC7277181; doi:10.3390/biom10050793)

Figure 1. The effect of VU-29 on short-time maintenance of ethanol-induced CPP in the CPP Test 1. Data are shown as post-conditioning minus pre-conditioning time (s) spent in the drug-associated compartment. Dots represent individual measurements, the central horizontal mark is the mean, and error bars represent SEM. ***P<0.001 vs. vehicle.

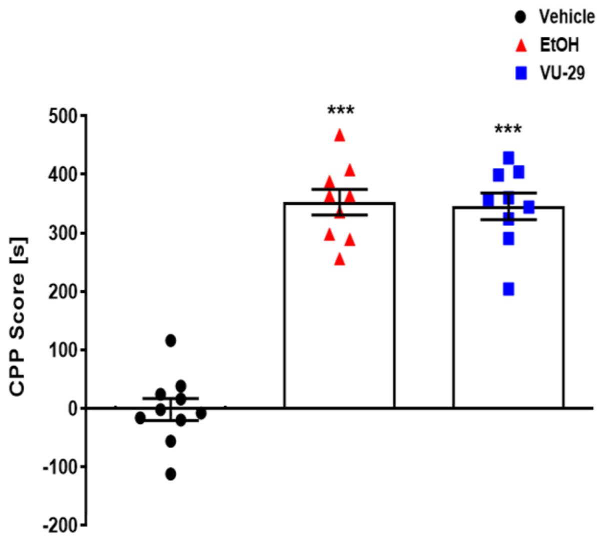

Figure 2. The effect of VU-29 administered in three different combinations on long-time maintenance of ethanol-induced CPP in the CPP Test 1. Data are shown as post-conditioning minus pre-conditioning time (s) spent in the drug-associated compartment. Dots represent individual measurements, the central horizontal mark is the mean, and error bars represent SEM. ***P<0.001 vs. vehicle.

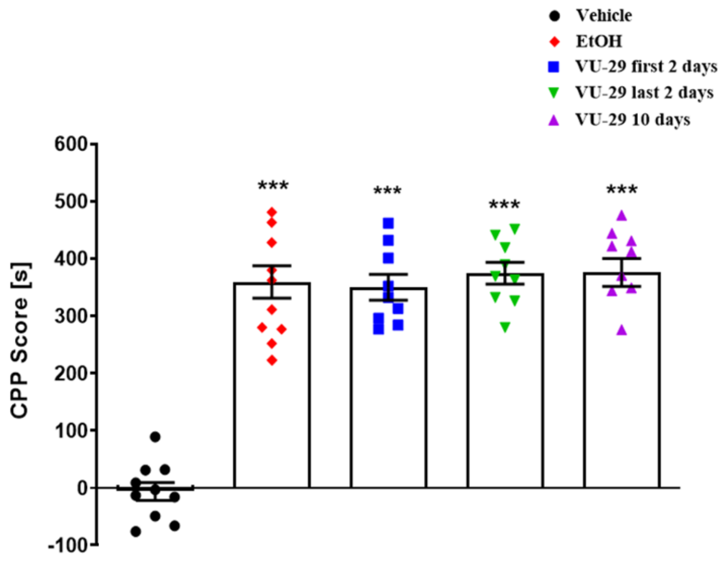

Figure 3. The influence of MK-801 and MTEP on the effect of VU-29 on the maintenance of ethanol-induced CPP in the CPP Test 1 (A). The influence of MK-801 and MTEP on CPP score in vehicle-treated rats in the CPP Test 1 (B). Data are shown as post-conditioning minus pre-conditioning time (s) spent in the drug-associated compartment. Dots represent individual measurements, the central horizontal mark is the mean, and error bars represent SEM. ***P<0.001 vs. vehicle.

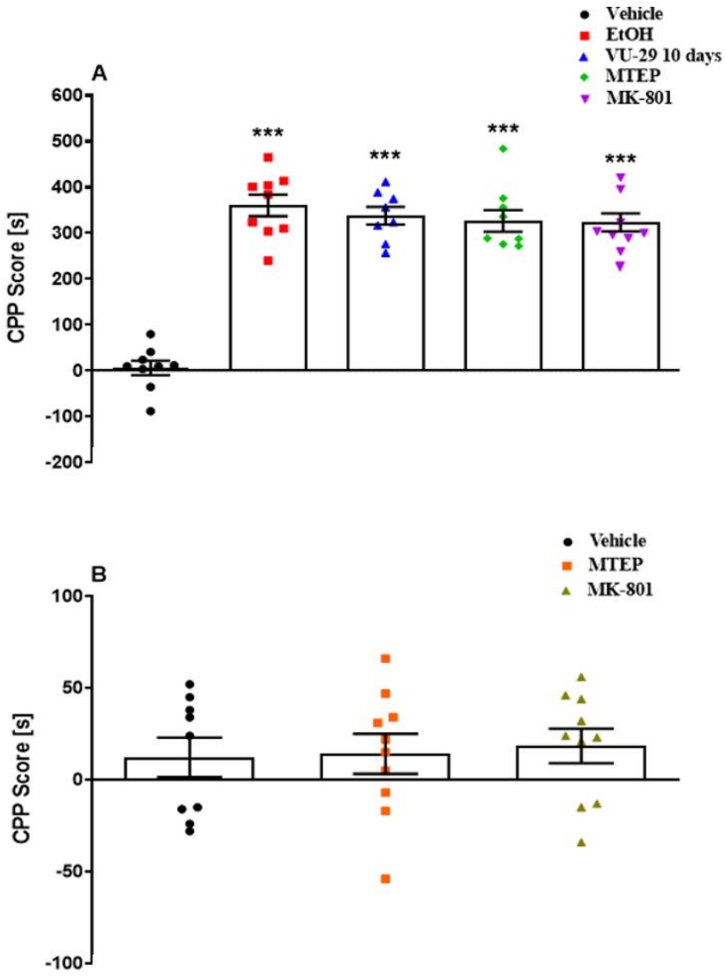

Supplement: Supplementary file 1 [file biomolecules-10-00793-s001.pdf]
